# Orthosilicates with glaserite-type crystal structures: Na_2_BaZr[SiO_4_]_2_ and Na_2_BaHf[SiO_4_]_2_

**DOI:** 10.1107/S2056989025002956

**Published:** 2025-04-04

**Authors:** Hisanori Yamane, Shiro Funahashi, Naoto Hirosaki, Takashi Takeda

**Affiliations:** a Research Center for Electronic and Optical Materials National Institute for Materials Science (NIMS) 1-1 Namiki Tsukuba Ibaraki 305-0044 Japan; Vienna University of Technology, Austria

**Keywords:** crystal structure, glaserite-type structure, orthosilicate, single-crystal X-ray diffraction, twin analysis

## Abstract

Single crystal particles of Na_2_BaZr[SiO_4_]_2_ [systematic name: disodium barium zirconium bis­(orthosilicate)] and Na_2_BaHf[SiO_4_]_2_ [disodium barium hafnium bis­(orthosilicate)] were extracted from grain-grown polycrystals obtained by heating compacts of binary oxide mixtures at 1473 K. Single crystal X-ray diffraction analysis revealed that these are isostructural orthosilicates with a glaserite-type crystal structure, in which all sites of *X*, *Y*, *M*, and *T* in the general formula *XY*_2_[*M*(*T*O_4_)_2_] are fully occupied by atoms of different elements. The crystal structures of the title compounds were refined in space group *P*3 under consideration of a two-component twin model. The SiO_4_ tetra­hedra are rotated approximately ±10.2° from the mirror plane of space group *P*3*m* around an axis parallel to [001].

## Abstract 2

The two title oxides are new isotypic silicates, crystallizing in the non-centrosymmetric space group *P*3. The crystal structures were analysed with two twin components in each case.

## 1. Chemical context

Nikolova & Kostov-Kytin (2013 ▸) described more than 100 oxides with glaserite-type crystal structures by the general formula *X*_(□;1)_*Y*_(□;2)_[*M*(*T*O_4_)_2_] and summarized their crystal structural features: the *T* sites are always fully occupied by the atoms of transition metals (V, Cr, Mo, W, Re, Fe, Ru) or non-metals (Si, P, S, Se). These *T* atoms are fourfold coordinated by oxygen atoms to form isolated tetra­hedra in the crystal structures. Silicates (*T* = Si), such as BaMg[SiO_4_] and Ba(Ba,Sr,Ca)_2_Mg[SiO_4_]_2_ doped with Eu^2+^, have been studied for their fluorescent properties and photochromism (Yonesaki *et al.*, 2008 ▸, 2011 ▸; Yonesaki, 2013 ▸; Yonezaki *et al.*, 2018 ▸; Yonezaki, 2015 ▸, 2018 ▸, 2020 ▸; Yonezaki & Takei, 2016 ▸; Yonezaki & Yanai, 2021 ▸; Birkel *et al.*, 2015 ▸). Recently, ferroaxial transitions of compounds with glaserite-type crystal structures were investigated, in which the space-group type changes from *P*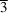 to *P*
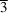
*m* (Yamagishi *et al.*, 2023 ▸). The rotation angle *φ* of the *T*O_4_ tetra­hedron was defined relative to the mirror plane of *P*
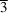
*m*, and BaCa_2_Mg[SiO_4_]_2_ with *φ =* 12.5° was proposed as a potential ferroaxial transition material. For the compounds with *M* = Zr, Kostov-Kytin and co-workers analysed the crystal structures of Na_3–*x*_H_1+*x*_Zr(SiO)_4_·*y*H_2_O in which water mol­ecules are located between the ZrO_6_ octa­hedra (Kostov-Kytin *et al.*, 2012 ▸, 2013 ▸).

In the current study, we report the synthesis and crystal structure analysis of two new orthosilicate compounds with glaserite-type crystal structure, Na_2_BaZr[SiO_4_]_2_ and Na_2_BaHf[SiO_4_]_2_.

## 2. Structural commentary

**Figure 1:**
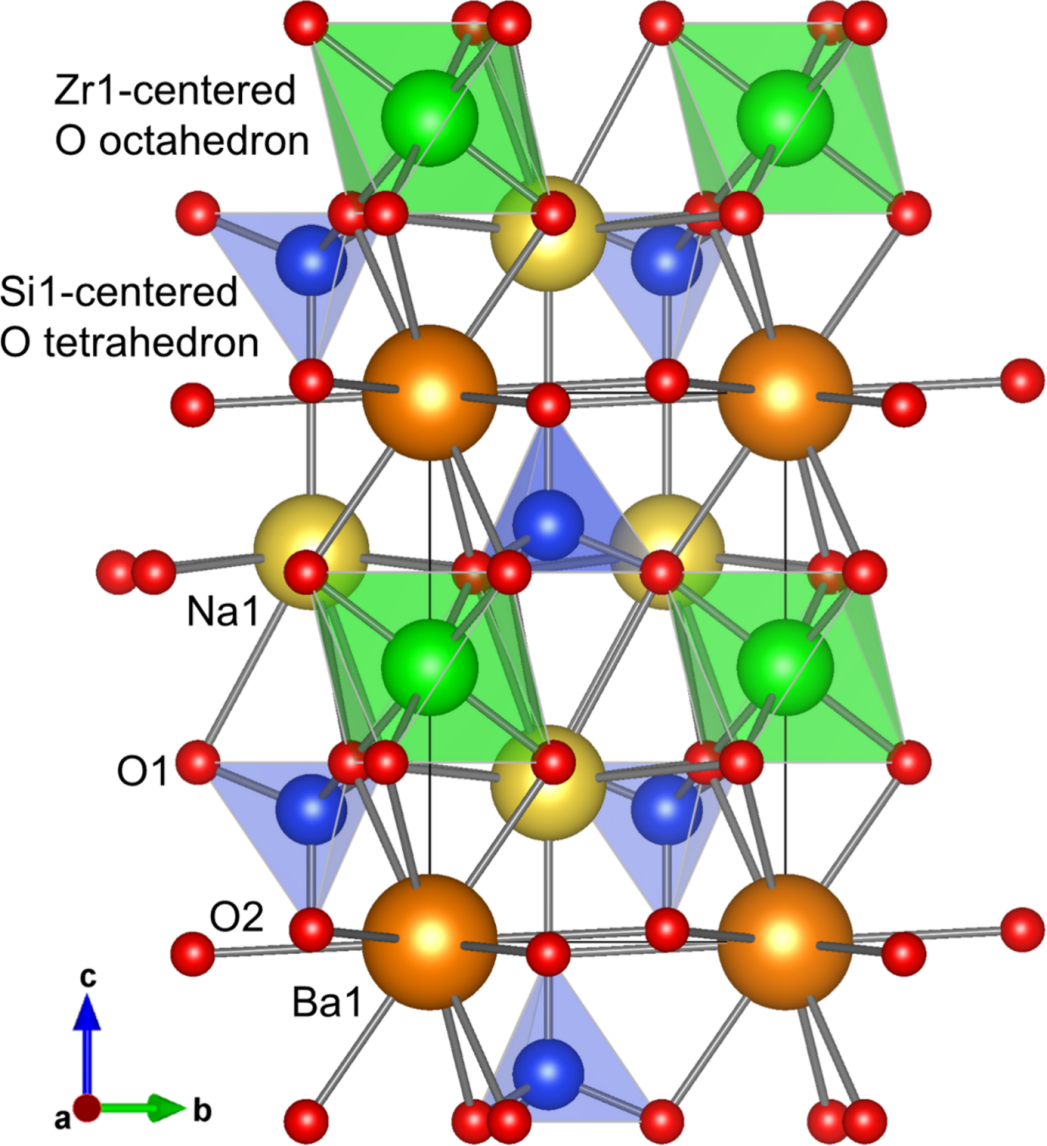
Crystal structure of Na_2_BaZr[SiO_4_]_2_ in a projection along [100], drawn with Zr1-centered oxygen octa­hedra and Si1-centered oxygen tetra­hedra.

According to the classification by Nikolova & Kostov-Kytin (2013 ▸) using the general formula *X*_(□;1)_*Y*_(□;2)_[*M*(*T*O_4_)_2_] for compounds with glaserite-type crystal structures, Na_2_BaZr[SiO_4_]_2_ and Na_2_BaHf[SiO_4_]_2_ meet the condition *X* ≠ *Y* ≠ *M* ≠ *T* of *XY*_2_[*M*(*T*O_4_)_2_] with no vacancy. The two new orthosilicates are isostructural and crystallize in the trigonal space group *P*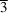. The crystal structures of both silicates were refined under consideration of a two-component twin model in each case. Multiplicity, Wyckoff letter, and site symmetry are: 1, *a* and 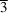 for Ba1, 1, *b* and 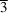 for Zr1/Hf1, 2, *d* and 3 for Na1, Si1 and O2, and 6, *g* and 1 for O1. As shown in Fig. 1 ▸, slabs identified in the crystal structure are composed of *M* = Zr- or Hf-centred oxygen octa­hedra and SiO_4_ tetra­hedra.

**Figure 2:**
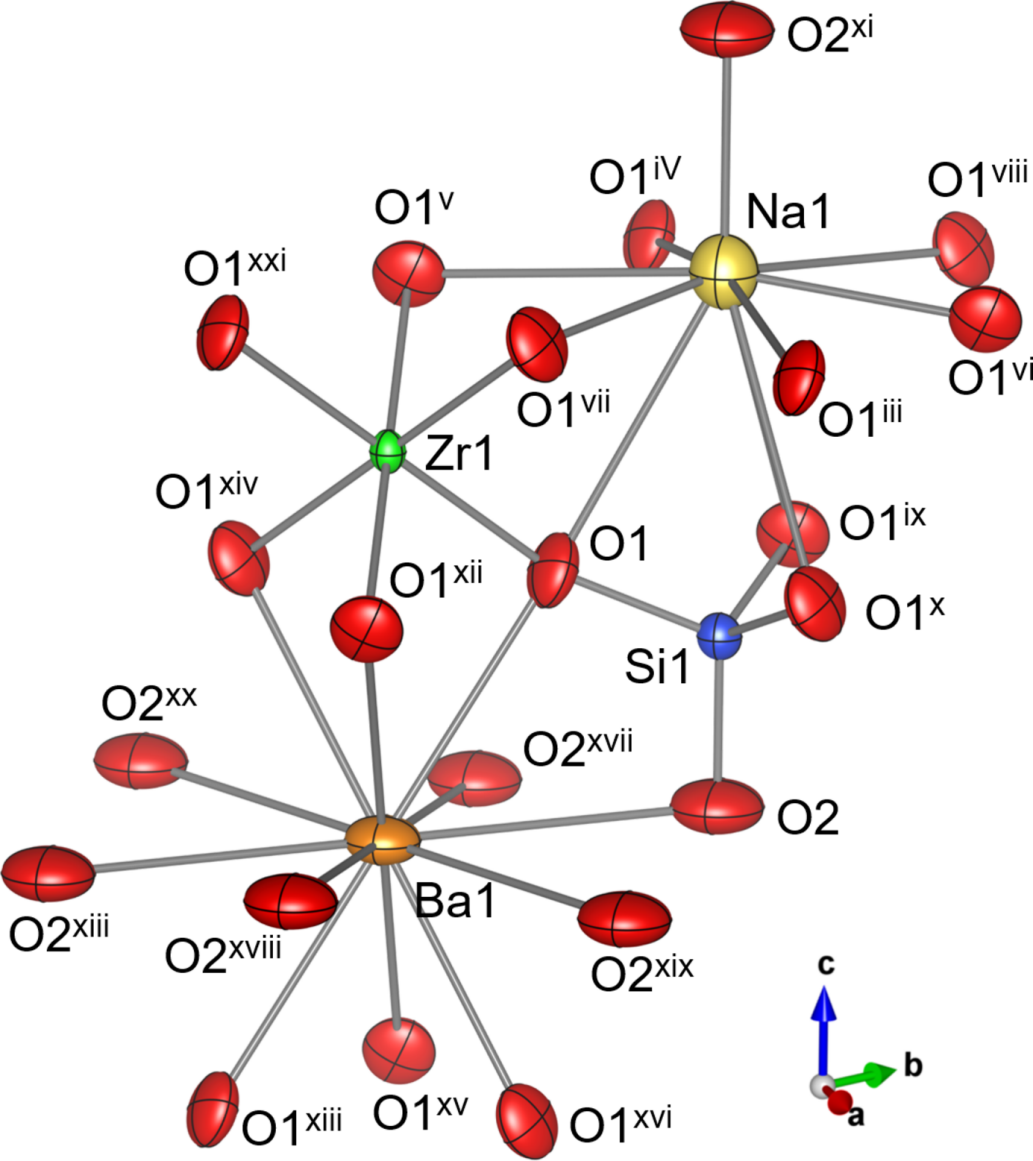
Atomic arrangements around Na1, Ba1, Zr1, and Si1 in the crystal structure of Na_2_BaZr[SiO_4_]_2_. Displacement ellipsoids are depicted at the 90% probability level. [Symmetry codes: (i) *x*, *y* + 1, *z*; (ii) −*x* + 1, −*y* + 2, −*z* + 1; (iii) −*x* + 1, −*y* + 1, −*z* + 1; (iv) −*x*, −*y* + 1, −*z* + 1; (v) *x* − *y*, *x*, −*z* + 1; (vi) *x* − *y* + 1, *x* + 1, −*z* + 1; (vii) *y*, −*x* + *y*, −*z* + 1; (viii) *y*, −*x* + *y* + 1, −*z* + 1; (ix) −*x* + *y*, −*x* + 1, *z*; (*x*) −*y* + 1, *x* − *y* + 1, *z*; (xi) *x*, *y*, *z* + 1; (xii) −*x* + *y*, −*x*, *z*; (xiii) −*x*, −*y*, −*z*; (xiv) −*y*, *x* − *y*, *z*; (xv) *x* − *y*, *x*, −*z*; (xvi) *y*, −*x* + *y*, −*z*; (xvii) −*x*, −*y* + 1, −*z*; (xviii) *x*, *y* − 1, *z*; (xix) −*x* + 1, −*y* + 1, −*z*; (xx) *x* − 1, *y* − 1, *z*; (xxi) −*x*, −*y*, −*z* + 1; (xxii) *x* + 1, *y* + 1, *z*; (xxiii) *x*, *y*, *z* − 1.]

**Figure 3:**
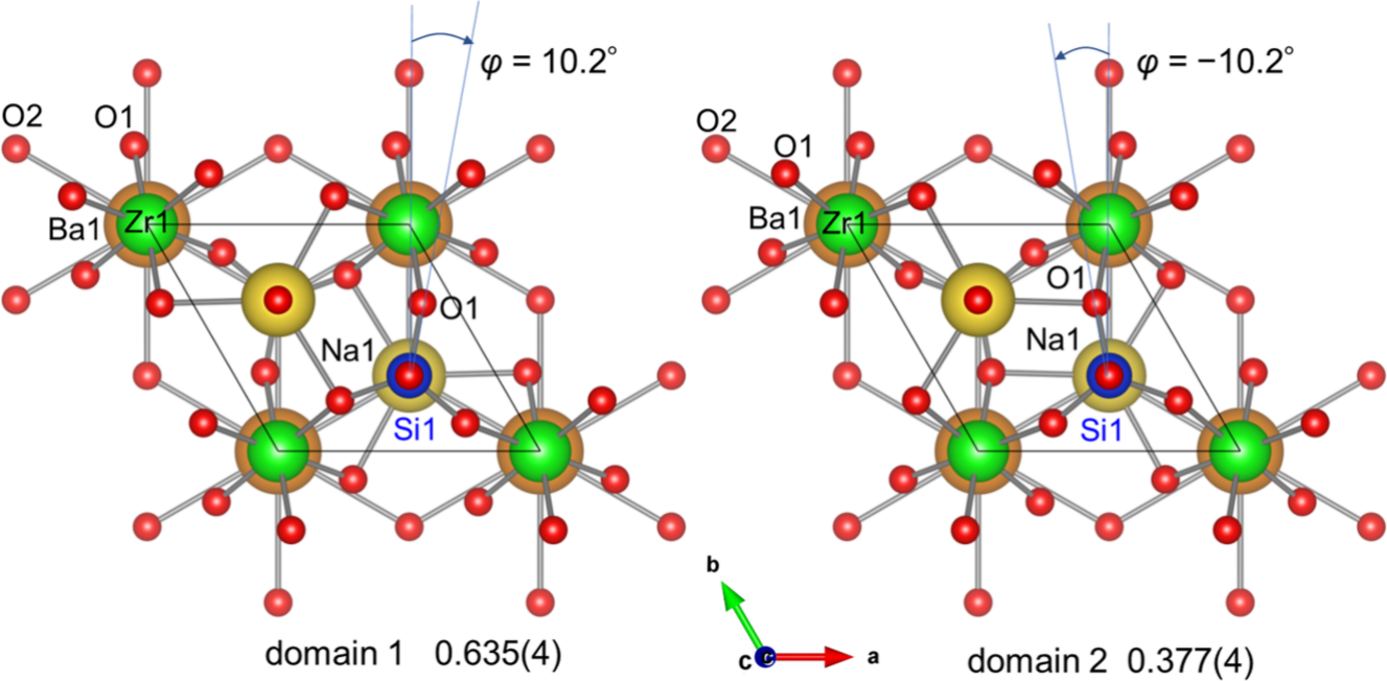
[001] projection of the twin domains in the crystal structure of Na_2_BaZr[SiO_4_]_2_.

Fig. 2 ▸ shows the arrangement of oxygen atoms around each cation. Inter­atomic distances of Zr—O and Hf—O are 2.0667 (14) Å (×6) and 2.0600 (15) Å (×6), respectively (Tables 1 ▸ and 2 ▸), which are consistent with the sizes of Shannon’s effective ionic radii [six-coordinated Zr (0.72 Å) and Hf (0.71 Å); Shannon, 1976 ▸]. The Si—O distances of 1.6373 (13) and 1.578 (2) Å for Na_2_BaZr[SiO_4_]_2_ agree with those of 1.6354 (16) and 1.575 (2) Å for Na_2_BaHf[SiO_4_]_2_. The Ba—O distances for the 12-coordinate *X* site are slightly longer for Na_2_BaZr[SiO_4_]_2_ [2.8764 (15)–3.1198 (2) Å] than for Na_2_BaHf[SiO_4_]_2_ [2.8687 (16)–3.1158 (2) Å]. The distances between O1 and Na1 at the tenfold coordination sites are slightly longer for Na_2_Ba*M*[SiO_4_]_2_*M* = Zr [2.4461 (15)–3.1545 (19) Å] than for *M* = Hf [2.4423 (15)–3.145 (2) Å], while the Na1—O2 distance of 2.250 (3) Å for *M* = Zr is slightly shorter than that of 2.258 (3) Å for *M* = Hf. The bond-valence sums for Na1, Ba1, Zr1, Hf1, and Si1 calculated with the bond valence parameters provided by Gagné & Hawthorne (2015 ▸) are 1.00, 1.86, 4.03, and 4.11 valence units for Na_2_Ba*M*[SiO_4_]_2_*M* = Zr, and 1.01, 1.89, 4.05, and 4.09 valence units for *M* = Hf. The rotation angles *φ* of the [SiO_4_] tetra­hedra are 10.18° for *M* = Zr and 10.15° for *M* = Hf (Fig. 3 ▸).

The Madelung energy part of the lattice energies (*MAPLE*; Hoppe, 1995 ▸) of Na_2_Ba*M*[SiO_4_]_2_ calculated using *VESTA* (Momma & Izumi, 2011 ▸) are −50.170 mJ mol^−1^ (*M* = Zr) and −50.260 MJ mol^−1^ (*M* = Hf). These values are close to those of −49.880 MJ mol^−1^ and −49.880 mJ mol^−1^ with differences of 0.6% and 0.8%, respectively, as calculated from the equation BaO + *M*O_2_ + *α*-Na_2_Si_2_O_5_ = Na_2_Ba*M*[SiO_4_]_2_ using *MAPLE* values calculated from the crystal structure data for BaO (–3.510 MJ mol^−1^; Zollweg, 1955 ▸), ZrO_2_ (–12.740 MJ mol^−1^; Gualtieri *et al.*, 1996 ▸), HfO_2_ (–12.740 MJ mol^−1^; Pathak *et al.*, 2020 ▸) and *α*-Na_2_Si_2_O_5_ (–33.630 MJ mol^−1^; Pant & Cruickshank, 1968 ▸).

## 3. Database survey

The crystal structures listed for glaserite-type silicates in the ICSD database (ICSD, 2025 ▸) are the high-temperature phase of Ca_2_[SiO_4_] (*P*
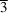
*m, Z* = 2, *V* = 194.19 Å^3^; Mumme *et al.*, 1996 ▸), Ba_3_Mg[SiO_4_]_2_ (*P*
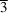
*m, Z* = 1, *V* = 198.64 Å^3^; Iwata *et al.*, 2009 ▸) and its superstructure (*P*
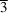
*, Z* = 3, *V* = 594.71 Å^3^; Park *et al.*, 2009 ▸), BaCa_2_Mg[SiO_4_]_2_ (*P*
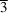
*, Z* = 1, *V* = 173.31 Å^3^; Park *et al.*, 2011 ▸), Ba_*x*_Sr_3–*x*_Mg[SiO_4_]_2_ (*x* = 0.0 – 0.5, *C*2, *Z* = 4, *V* = 714.9 – 723.7 Å^3^; *x* = 0.625 – 2.375, *P*
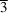
*m*, *Z* = 1, *V* = 181.81 – 193.46 Å^3^; *x* = 2.5 – 3.0, *P*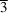, *Z* = 3, *V* = 583.2 – 594.72 Å^3^; Yonezaki, 2015 ▸), Ba(Sr_1-*x*_Ca_*x*_)_2_Mg[SiO_4_]_2_ (*x* = 0.0 – 0.5, *P*
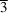
*m*, *Z* = 1, *V* = 182.76 – 178.70 Å^3^; *x* = 0.5625 – 1.0, *P*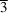, *Z* = 1, *V* = 177.97 – 174.00 Å^3^; Yonesaki *et al.*, 2008 ▸; Yonesaki, 2013 ▸), Ba_3_Mn[SiO_4_] (*P*
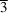
*m*, *Z* = 1, *V* = 203.44 Å^3^; Avdeev *et al.*, 2018 ▸), Na_3_HZr[SiO_4_]_2_, *P*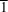, *Z* = 2, *V* = 350.12 Å^3^; Na_3_HZr[SiO_4_]_2_·0.4H_2_O, *P*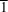, *Z* = 2, *V* = 350.19 Å^3^; Na_3_HZr[SiO_4_]_2_·H_2_O, *C*2/*m*, Z = 4, *V* = 683.92 Å^3^; Kostov-Kytin *et al.*, 2012 ▸).

## 4. Synthesis and crystallization

The starting materials were powders of Na_2_O (∼80%, Sigma-Aldrich), SiO_2_ (99.9% Kojundo Chemical Lab. Co., Ltd.), BaO (99.99%, Sigma-Aldrich), ZrO_2_ (99%, Sigma-Aldrich), and HfO_2_ (98%, Kojundo Chemical Lab. Co., Ltd.), which were weighed in a glove box in a nitro­gen atmosphere, mixed in an agate mortar, and formed into disk-shaped compacts. The compacts were placed in a nickel boat and sealed in a stainless-steel container. The container was heated up to 1473 K for 3 h in a nitro­gen gas flow to prevent oxidation of the stainless steel, and this temperature was maintained for 30 min. The temperature was subsequently lowered to 1073 K at a rate of −100 K h^−1^. The power supply to the heater wire was stopped at this temperature, and the sample was allowed to cool in the furnace. The single crystal grains of Na_2_BaZr[SiO_4_]_2_ used for single crystal X-ray diffraction data measurements were isolated from fragments of polycrystals synthesised from a starting material mixture with a metal element molar ratio of Na:Ba:Zr:Si = 2:1:1:2. Single crystal grains of Na_2_BaHf[SiO_4_]_2_ were obtained from polycrystals prepared with a starting mixture of Na:Ba:Hf:Si = 2.2:1:1:2.2.

Semi-qu­anti­tative analysis of the Na_2_BaZr[SiO_4_]_2_ and Na_2_BaHf[SiO_4_]_2_ grains was performed using an energy-dispersive X-ray detector (Bruker AXS, XFlash 5010) attached to a scanning electron microscope (Hitachi High-Tech SU1510) and measurement analysis software (Bruker AXS, QUANTAX2000). The atomic ratios acquired from the analysis were Na:Ba:Hf:Si = 2.0 (3):0.6 (1):0.66 (5):2.0 (3) for Na_2_BaZr[SiO_4_]_2_ and Na:Ba:Zr:Si = 2.3 (4):1.2 (1):1.3 (1):2.0 (1) for Na_2_BaHf[SiO_4_]_2_, both of which correspond sufficiently with the metal element ratios of the formulae.

## 5. Refinement

Crystal data, data collection and structural refinement details are summarised in Table 3 ▸. In the first refinement step, the ideal model of the glaserite-type structure with space group *P*
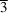
*m* was adopted and the *R*1 values were 0.032 and 0.018 for Na_2_Ba*M*[SiO_4_]_2_ for *M* = Zr and Hf, respectively. However, the ratio of the mean-square displacements of the major and minor axes of the atomic displacement ellipsoid of O1 was 30.3 (*M* = Zr) and 22.4 *(M* = Hf). Subsequently, when the refinement was performed in *P*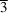 removing mirror symmetry operation from *P*
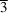
*m*, the *R*1 values were 0.035 and 0.020, and the displacement ratios were still large at 27.6 and 18.2, for *M* = Zr and Hf, respectively. Further analysis in space group *P*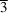 under consideration of twinning (twin matrix 010, 100, 00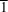) resulted in *R*1 values of 0.016 and 0.010 for *M* = Zr and Hf, respectively, and both displacement ratios were reasonable with a value of 3.2. The twin ratios of domains 1 and 2 were refined to 0.635 (4):0.365 for *M* = Zr and 0.623 (4):0.377 (4) for *M* = Hf.

## Supplementary Material

Crystal structure: contains datablock(s) global, I, II. DOI: 10.1107/S2056989025002956/wm5753sup1.cif

Structure factors: contains datablock(s) I. DOI: 10.1107/S2056989025002956/wm5753Isup2.hkl

Structure factors: contains datablock(s) II. DOI: 10.1107/S2056989025002956/wm5753IIsup3.hkl

CCDC references: 2440344, 2440343

Additional supporting information:  crystallographic information; 3D view; checkCIF report

## Figures and Tables

**Table 1:** Selected bond lengths (Å) for Na_2_BaZr[SiO_4_]_2_

Na1—O1^i^	2.9791 (15)	Ba1—O2	3.1198 (2)
Na1—O1^ii^	2.4461 (15)	Zr1—O1	2.0667 (13)
Na1—O1	3.1545 (19)	Si1—O1	1.6373 (13)
Na1—O2^iii^	2.250 (3)	Si1—O2	1.578 (2)
Ba1—O1	2.8764 (15)		

Symmetry codes: (i) 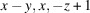; (ii) 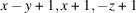; (iii) 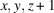.

**Table 2:** Selected bond lengths (Å) for Na_2_BaHf[SiO_4_]_2_

Na1—O1^i^	2.4423 (15)	Ba1—O2	3.1158 (2)
Na1—O1^ii^	2.9738 (15)	Hf1—O1	2.0600 (15)
Na1—O1	3.145 (2)	Si1—O1	1.6354 (16)
Na1—O2^iii^	2.258 (3)	Si1—O2	1.575 (2)
Ba1—O1	2.8687 (16)		

Symmetry codes: (i) 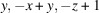; (ii) 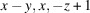; (iii) 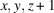.

**Table 3:** Experimental details

	Na_2_BaZr[SiO_4_]_2_	Na_2_BaHf[SiO_4_]_2_
Crystal data		
*M*_r_	458.72	545.99
Crystal system, space group	Trigonal, *P* 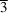	Trigonal, *P* 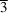
Temperature (K)	293	293
*a*, *c* (Å)	5.3966 (2), 7.2153 (3)	5.3889 (2), 7.1996 (2)
*V* (Å^3^)	181.98 (2)	181.07 (1)
*Z*	1	1
Radiation type	Mo *K*α	Mo *K*α
μ (mm^−1^)	7.27	20.19
Crystal size (mm)	0.04 × 0.03 × 0.01	0.03 × 0.03 × 0.01

Data collection		
Diffractometer	ROD, Synergy Custom system, HyPix-Arc 150	ROD, Synergy Custom system, HyPix-Arc 150
Absorption correction	Gaussian (*CrysAlis PRO*; Rigaku OD, 2023 ▸)	Gaussian (*CrysAlis PRO*; Rigaku OD, 2023 ▸)
*T*_min_, *T*_max_	0.544, 0.817	0.641, 0.883
No. of measured, independent and observed [*I* > 2σ(*I*)] reflections	9034, 587, 543	8609, 538, 532
*R*_int_	0.043	0.045
(sin θ/λ)_max_ (Å^−1^)	0.832	0.806

Refinement		
*R*[*F*^2^ > 2σ(*F*^2^)], *wR*(*F*^2^), *S*	0.016, 0.035, 1.11	0.010, 0.024, 1.11
No. of reflections	587	538
No. of parameters	24	24
Δρ_max_, Δρ_min_ (e Å^−3^)	0.52, −0.59	0.61, −0.60

Computer programs: *CrysAlis PRO* (Rigaku OD, 2023 ▸), *SHELXT* (Sheldrick, 2015*a* ▸), *SHELXL* (Sheldrick, 2015*b* ▸), *VESTA* (Momma & Izumi, 2011 ▸), *OLEX2* (Dolomanov *et al.*, 2009 ▸) and *publCIF* (Westrip, 2010 ▸).

## supplementary crystallographic information

### Disodium barium zirconium bis(orthosilicate) (I) Crystal data

**Table:**

Na_2_BaZr[SiO_4_]_2_	*D*_x_ = 4.186 Mg m^−^^3^
*M**_r_* = 458.72	Mo *K*α radiation, λ = 0.71073 Å
Trigonal, *P*3	Cell parameters from 5734 reflections
*a* = 5.3966 (2) Å	θ = 2.8–47.6°
*c* = 7.2153 (3) Å	µ = 7.27 mm^−^^1^
*V* = 181.98 (2) Å^3^	*T* = 293 K
*Z* = 1	Plate, colourless
*F*(000) = 210	0.04 × 0.03 × 0.01 mm

### Disodium barium zirconium bis(orthosilicate) (I) Data collection

**Table:**

ROD, Synergy Custom system, HyPix-Arc 150 diffractometer	587 independent reflections
Radiation source: Rotating-anode X-ray tube, Rigaku (Mo) X-ray Source	543 reflections with *I* > 2σ(*I*)
Mirror monochromator	*R*_int_ = 0.043
Detector resolution: 10.0000 pixels mm^-1^	θ_max_ = 36.2°, θ_min_ = 2.8°
ω scans	*h* = −8→7
Absorption correction: gaussian (CrysAlisPro; Rigaku OD, 2023)	*k* = −8→8
*T*_min_ = 0.544, *T*_max_ = 0.817	*l* = −12→12
9034 measured reflections	

### Disodium barium zirconium bis(orthosilicate) (I) Refinement

**Table:**

Refinement on *F*^2^	0 restraints
Least-squares matrix: full	Primary atom site location: dual
*R*[*F*^2^ > 2σ(*F*^2^)] = 0.016	*w* = 1/[σ^2^(*F*_o_^2^) + (0.0092*P*)^2^ + 0.1709*P*] where *P* = (*F*_o_^2^ + 2*F*_c_^2^)/3
*wR*(*F*^2^) = 0.035	(Δ/σ)_max_ < 0.001
*S* = 1.11	Δρ_max_ = 0.52 e Å^−^^3^
587 reflections	Δρ_min_ = −0.59 e Å^−^^3^
24 parameters	

### Disodium barium zirconium bis(orthosilicate) (I) Special details

**Table:**

Geometry. All esds (except the esd in the dihedral angle between two l.s. planes) are estimated using the full covariance matrix. The cell esds are taken into account individually in the estimation of esds in distances, angles and torsion angles; correlations between esds in cell parameters are only used when they are defined by crystal symmetry. An approximate (isotropic) treatment of cell esds is used for estimating esds involving l.s. planes.
Refinement. Refined as a 2-component twin. 1. Twinned data refinement Scales: 0.635 (4) 0.365 (4)

### Disodium barium zirconium bis(orthosilicate) (I) Fractional atomic coordinates and isotropic or equivalent isotropic displacement parameters (Å^2^)

**Table:**

	*x*	*y*	*z*	*U*_iso_*/*U*_eq_	
Na1	0.333333	0.666667	0.71034 (19)	0.0167 (2)	
Ba1	0.000000	0.000000	0.000000	0.01522 (7)	
Zr1	0.000000	0.000000	0.500000	0.00498 (6)	
Si1	0.333333	0.666667	0.24086 (9)	0.00685 (12)	
O1	0.1242 (3)	0.3476 (3)	0.3269 (2)	0.0144 (3)	
O2	0.333333	0.666667	0.0221 (3)	0.0222 (5)	

### Disodium barium zirconium bis(orthosilicate) (I) Atomic displacement parameters (Å^2^)

**Table:**

	*U*^11^	*U*^22^	*U*^33^	*U*^12^	*U*^13^	*U*^23^
Na1	0.0157 (4)	0.0157 (4)	0.0188 (6)	0.00784 (18)	0.000	0.000
Ba1	0.01899 (9)	0.01899 (9)	0.00767 (9)	0.00950 (4)	0.000	0.000
Zr1	0.00436 (8)	0.00436 (8)	0.00620 (12)	0.00218 (4)	0.000	0.000
Si1	0.00661 (17)	0.00661 (17)	0.0073 (3)	0.00331 (9)	0.000	0.000
O1	0.0154 (7)	0.0082 (5)	0.0175 (6)	0.0043 (5)	0.0010 (5)	0.0046 (4)
O2	0.0287 (8)	0.0287 (8)	0.0093 (8)	0.0144 (4)	0.000	0.000

### Disodium barium zirconium bis(orthosilicate) (I) Geometric parameters (Å, º)

**Table:**

Na1—Zr1^i^	3.4657 (6)	Ba1—O1^xvi^	2.8763 (15)
Na1—Si1^ii^	3.1356 (2)	Ba1—O1	2.8763 (15)
Na1—Si1^iii^	3.1356 (2)	Ba1—O2^xvii^	3.1198 (2)
Na1—Si1	3.3875 (15)	Ba1—O2	3.1198 (2)
Na1—Si1^iv^	3.1356 (2)	Ba1—O2^xviii^	3.1198 (2)
Na1—O1^v^	2.9791 (15)	Ba1—O2^xix^	3.1198 (2)
Na1—O1^vi^	2.4461 (15)	Ba1—O2^xiii^	3.1198 (2)
Na1—O1^iv^	2.4461 (14)	Ba1—O2^xx^	3.1198 (2)
Na1—O1^vii^	2.4461 (14)	Zr1—O1^xxi^	2.0666 (13)
Na1—O1^viii^	2.9791 (15)	Zr1—O1^vii^	2.0666 (14)
Na1—O1^iii^	2.9791 (15)	Zr1—O1	2.0667 (13)
Na1—O1	3.1545 (19)	Zr1—O1^v^	2.0667 (13)
Na1—O1^ix^	3.1545 (19)	Zr1—O1^xiv^	2.0666 (14)
Na1—O1^x^	3.1545 (19)	Zr1—O1^xii^	2.0667 (13)
Na1—O2^xi^	2.250 (3)	Si1—O1^x^	1.6373 (13)
Ba1—O1^xii^	2.8764 (15)	Si1—O1^ix^	1.6373 (13)
Ba1—O1^xiii^	2.8763 (15)	Si1—O1	1.6373 (13)
Ba1—O1^xiv^	2.8763 (15)	Si1—O2	1.578 (2)
Ba1—O1^xv^	2.8764 (15)		
			
Si1^iii^—Na1—Zr1^i^	160.48 (5)	Na1—Zr1—Na1^iii^	77.74 (2)
Si1^iv^—Na1—Zr1^i^	66.579 (9)	Na1—Zr1—Na1^xxi^	180.0
Si1^ii^—Na1—Zr1^i^	66.579 (9)	Na1^iii^—Zr1—Na1^xx^	180.0
Si1—Na1—Zr1^i^	64.03 (2)	Na1^iv^—Zr1—Na1^iii^	102.26 (2)
Si1^iii^—Na1—Si1	96.45 (3)	Na1^xviii^—Zr1—Na1^iv^	180.0
Si1^iv^—Na1—Si1^iii^	118.756 (11)	Na1^xxi^—Zr1—Na1^iii^	102.26 (2)
Si1^iv^—Na1—Si1	96.45 (3)	Na1^xviii^—Zr1—Na1^xxi^	77.74 (2)
Si1^ii^—Na1—Si1^iv^	118.757 (11)	Na1^iv^—Zr1—Na1	77.74 (2)
Si1^ii^—Na1—Si1^iii^	118.756 (11)	Na1^xviii^—Zr1—Na1^xx^	102.26 (2)
Si1^ii^—Na1—Si1	96.45 (3)	Na1^xviii^—Zr1—Na1^iii^	77.74 (2)
O1^iii^—Na1—Zr1^i^	138.23 (4)	O1—Zr1—Na1^xviii^	135.97 (4)
O1^vii^—Na1—Zr1^i^	137.54 (5)	O1^vii^—Zr1—Na1^xviii^	58.86 (4)
O1^iv^—Na1—Zr1^i^	35.96 (3)	O1^xxi^—Zr1—Na1^xx^	58.86 (4)
O1^viii^—Na1—Zr1^i^	36.43 (3)	O1^vii^—Zr1—Na1^iii^	63.77 (5)
O1^v^—Na1—Zr1^i^	86.58 (3)	O1^xii^—Zr1—Na1^xx^	135.97 (4)
O1^vi^—Na1—Zr1^i^	85.84 (4)	O1^xxi^—Zr1—Na1	116.23 (5)
O1^iv^—Na1—Si1	83.70 (5)	O1^v^—Zr1—Na1^xx^	44.03 (4)
O1^vi^—Na1—Si1	83.70 (5)	O1^v^—Zr1—Na1^xviii^	116.23 (5)
O1^vii^—Na1—Si1^iii^	31.10 (3)	O1^v^—Zr1—Na1^iii^	135.97 (4)
O1^vii^—Na1—Si1	83.70 (5)	O1^xii^—Zr1—Na1^iii^	44.03 (4)
O1^iii^—Na1—Si1	84.83 (4)	O1^xiv^—Zr1—Na1^xviii^	121.14 (4)
O1^v^—Na1—Si1	84.83 (4)	O1^xii^—Zr1—Na1^xviii^	63.77 (5)
O1^v^—Na1—Si1^iii^	91.85 (3)	O1^xiv^—Zr1—Na1^iv^	58.86 (4)
O1^iii^—Na1—Si1^iv^	148.85 (3)	O1—Zr1—Na1^iv^	44.03 (4)
O1^v^—Na1—Si1^ii^	148.85 (3)	O1^xxi^—Zr1—Na1^xviii^	44.03 (4)
O1^viii^—Na1—Si1	84.83 (4)	O1^xii^—Zr1—Na1	121.14 (4)
O1^iii^—Na1—Si1^ii^	91.85 (3)	O1^v^—Zr1—Na1^iv^	63.77 (5)
O1^vii^—Na1—Si1^ii^	148.63 (3)	O1—Zr1—Na1^iii^	58.86 (4)
O1^iv^—Na1—Si1^iv^	31.10 (3)	O1—Zr1—Na1^xxi^	116.23 (5)
O1^viii^—Na1—Si1^iii^	148.85 (3)	O1^xxi^—Zr1—Na1^iv^	135.97 (4)
O1^vi^—Na1—Si1^iv^	148.63 (3)	O1^v^—Zr1—Na1^xxi^	121.14 (4)
O1^viii^—Na1—Si1^ii^	30.93 (3)	O1^xii^—Zr1—Na1^iv^	116.23 (5)
O1^vii^—Na1—Si1^iv^	92.26 (3)	O1^xii^—Zr1—Na1^xxi^	58.86 (4)
O1^iv^—Na1—Si1^ii^	92.26 (3)	O1^vii^—Zr1—Na1^xx^	116.23 (5)
O1^viii^—Na1—Si1^iv^	91.85 (3)	O1^vii^—Zr1—Na1^iv^	121.14 (4)
O1^iii^—Na1—Si1^iii^	30.93 (3)	O1—Zr1—Na1^xx^	121.14 (4)
O1^v^—Na1—Si1^iv^	30.93 (3)	O1^xxi^—Zr1—Na1^xxi^	63.77 (5)
O1^iv^—Na1—Si1^iii^	148.63 (3)	O1^xiv^—Zr1—Na1^xx^	63.77 (5)
O1^vi^—Na1—Si1^ii^	31.10 (3)	O1^vii^—Zr1—Na1	44.03 (4)
O1^vi^—Na1—Si1^iii^	92.26 (3)	O1^xxi^—Zr1—Na1^iii^	121.14 (4)
O1^vii^—Na1—O1^iii^	56.84 (6)	O1^xiv^—Zr1—Na1	135.97 (4)
O1^vi^—Na1—O1^viii^	56.84 (6)	O1^vii^—Zr1—Na1^xxi^	135.97 (4)
O1^iv^—Na1—O1^vii^	118.811 (19)	O1^v^—Zr1—Na1	58.86 (4)
O1^iv^—Na1—O1^viii^	62.52 (6)	O1^xiv^—Zr1—Na1^iii^	116.23 (5)
O1^vi^—Na1—O1^iii^	62.52 (6)	O1^xiv^—Zr1—Na1^xxi^	44.03 (4)
O1^vi^—Na1—O1^v^	168.18 (9)	O1—Zr1—Na1	63.77 (5)
O1^v^—Na1—O1^iii^	119.197 (12)	O1^xxi^—Zr1—O1^xii^	92.75 (6)
O1^vii^—Na1—O1^v^	62.52 (6)	O1^xiv^—Zr1—O1	87.25 (6)
O1^iv^—Na1—O1^v^	56.84 (6)	O1^xxi^—Zr1—O1^xiv^	92.75 (6)
O1^vii^—Na1—O1^viii^	168.18 (9)	O1^xxi^—Zr1—O1	180.0
O1^iv^—Na1—O1^vi^	118.810 (18)	O1^xxi^—Zr1—O1^vii^	87.25 (6)
O1^viii^—Na1—O1^iii^	119.197 (13)	O1^xiv^—Zr1—O1^v^	92.75 (6)
O1^viii^—Na1—O1^v^	119.196 (13)	O1—Zr1—O1^xii^	87.25 (6)
O1^vi^—Na1—O1^vii^	118.812 (19)	O1^vii^—Zr1—O1^xii^	92.75 (6)
O1^iv^—Na1—O1^iii^	168.18 (9)	O1^vii^—Zr1—O1^v^	87.25 (6)
O2^xi^—Na1—Zr1^i^	115.97 (2)	O1^xxi^—Zr1—O1^v^	87.25 (6)
O2^xi^—Na1—Si1	180.0	O1—Zr1—O1^v^	92.75 (6)
O2^xi^—Na1—Si1^iv^	83.55 (3)	O1^xiv^—Zr1—O1^xii^	87.25 (6)
O2^xi^—Na1—Si1^iii^	83.55 (3)	O1^vii^—Zr1—O1	92.75 (6)
O2^xi^—Na1—Si1^ii^	83.55 (3)	O1^vii^—Zr1—O1^xiv^	180.0
O2^xi^—Na1—O1^v^	95.17 (4)	O1^v^—Zr1—O1^xii^	180.0
O2^xi^—Na1—O1^iv^	96.30 (5)	Na1^iii^—Si1—Na1	83.55 (3)
O2^xi^—Na1—O1^vi^	96.30 (5)	Na1^ii^—Si1—Na1^iii^	118.756 (11)
O2^xi^—Na1—O1^vii^	96.30 (5)	Na1^ii^—Si1—Na1	83.55 (3)
O2^xi^—Na1—O1^viii^	95.17 (4)	Na1^iv^—Si1—Na1	83.55 (3)
O2^xi^—Na1—O1^iii^	95.17 (4)	Na1^iv^—Si1—Na1^iii^	118.756 (11)
O1—Ba1—O1^xiv^	59.43 (4)	Na1^ii^—Si1—Na1^iv^	118.757 (11)
O1^xiii^—Ba1—O1^xv^	59.43 (4)	Na1^ii^—Si1—Ba1^i^	67.716 (15)
O1—Ba1—O1^xvi^	120.57 (4)	Na1^iv^—Si1—Ba1	67.715 (15)
O1—Ba1—O1^xii^	59.43 (4)	Na1^ii^—Si1—Ba1	157.30 (3)
O1^xiii^—Ba1—O1^xiv^	120.57 (4)	Na1^iv^—Si1—Ba1^xxii^	157.30 (3)
O1^xiv^—Ba1—O1^xii^	59.43 (4)	Na1^iv^—Si1—Ba1^i^	67.716 (15)
O1^xvi^—Ba1—O1^xiv^	180.00 (10)	Na1—Si1—Ba1	119.151 (10)
O1^xvi^—Ba1—O1^xii^	120.57 (4)	Na1^iii^—Si1—Ba1^xxii^	67.715 (15)
O1^xiii^—Ba1—O1	180.0	Na1^iii^—Si1—Ba1^i^	157.30 (3)
O1^xiii^—Ba1—O1^xii^	120.57 (4)	Na1^iii^—Si1—Ba1	67.715 (15)
O1^xiii^—Ba1—O1^xvi^	59.43 (4)	Na1—Si1—Ba1^xxii^	119.151 (10)
O1—Ba1—O1^xv^	120.57 (4)	Na1^ii^—Si1—Ba1^xxii^	67.715 (15)
O1^xv^—Ba1—O1^xii^	180.00 (10)	Na1—Si1—Ba1^i^	119.151 (10)
O1^xiv^—Ba1—O1^xv^	120.57 (4)	Ba1—Si1—Ba1^xxii^	98.284 (12)
O1^xvi^—Ba1—O1^xv^	59.43 (4)	Ba1^i^—Si1—Ba1	98.284 (12)
O1^xiv^—Ba1—O2^xix^	127.30 (5)	Ba1^i^—Si1—Ba1^xxii^	98.284 (12)
O1^xiv^—Ba1—O2^xvii^	80.82 (4)	O1^x^—Si1—Na1^iii^	50.51 (5)
O1^xvi^—Ba1—O2^xix^	52.70 (5)	O1—Si1—Na1^iii^	69.25 (5)
O1^xii^—Ba1—O2	99.18 (4)	O1^ix^—Si1—Na1^iii^	149.59 (7)
O1^xv^—Ba1—O2^xiii^	99.18 (4)	O1^ix^—Si1—Na1^iv^	69.25 (5)
O1—Ba1—O2^xix^	80.82 (4)	O1^ix^—Si1—Na1^ii^	50.51 (5)
O1^xiii^—Ba1—O2^xx^	80.82 (4)	O1—Si1—Na1	67.72 (6)
O1^xii^—Ba1—O2^xix^	71.30 (5)	O1^x^—Si1—Na1	67.72 (6)
O1^xvi^—Ba1—O2^xiii^	108.70 (5)	O1^x^—Si1—Na1^ii^	69.25 (5)
O1^xiv^—Ba1—O2^xiii^	71.30 (5)	O1—Si1—Na1^ii^	149.59 (7)
O1^xiii^—Ba1—O2^xix^	99.18 (4)	O1—Si1—Na1^iv^	50.51 (5)
O1—Ba1—O2^xx^	99.18 (4)	O1^x^—Si1—Na1^iv^	149.59 (7)
O1^xv^—Ba1—O2^xix^	108.70 (5)	O1^ix^—Si1—Na1	67.72 (6)
O1^xvi^—Ba1—O2	71.30 (5)	O1—Si1—Ba1^xxii^	134.92 (5)
O1^xvi^—Ba1—O2^xx^	127.30 (5)	O1—Si1—Ba1^i^	117.30 (5)
O1^xiv^—Ba1—O2^xx^	52.70 (5)	O1—Si1—Ba1	52.35 (6)
O1^xv^—Ba1—O2	80.82 (4)	O1^ix^—Si1—Ba1^i^	52.36 (6)
O1—Ba1—O2^xiii^	127.30 (5)	O1^x^—Si1—Ba1	117.30 (5)
O1^xiv^—Ba1—O2^xviii^	99.18 (4)	O1^ix^—Si1—Ba1^xxii^	117.30 (5)
O1^xii^—Ba1—O2^xx^	108.70 (5)	O1^ix^—Si1—Ba1	134.92 (5)
O1^xv^—Ba1—O2^xviii^	127.30 (5)	O1^x^—Si1—Ba1^xxii^	52.35 (6)
O1^xii^—Ba1—O2^xiii^	80.82 (4)	O1^x^—Si1—Ba1^i^	134.92 (5)
O1^xii^—Ba1—O2^xviii^	52.70 (5)	O1^ix^—Si1—O1	106.53 (6)
O1^xii^—Ba1—O2^xvii^	127.30 (5)	O1—Si1—O1^x^	106.53 (6)
O1^xiii^—Ba1—O2	127.30 (5)	O1^ix^—Si1—O1^x^	106.53 (6)
O1—Ba1—O2	52.70 (5)	O2—Si1—Na1	180.0
O1^xiii^—Ba1—O2^xvii^	108.70 (5)	O2—Si1—Na1^ii^	96.45 (3)
O1^xiii^—Ba1—O2^xviii^	71.30 (5)	O2—Si1—Na1^iv^	96.45 (3)
O1—Ba1—O2^xvii^	71.30 (5)	O2—Si1—Na1^iii^	96.45 (3)
O1^xiv^—Ba1—O2	108.70 (5)	O2—Si1—Ba1	60.849 (10)
O1^xvi^—Ba1—O2^xvii^	99.18 (4)	O2—Si1—Ba1^xxii^	60.849 (10)
O1—Ba1—O2^xviii^	108.70 (5)	O2—Si1—Ba1^i^	60.849 (10)
O1^xv^—Ba1—O2^xvii^	52.70 (5)	O2—Si1—O1	112.28 (6)
O1^xiii^—Ba1—O2^xiii^	52.70 (5)	O2—Si1—O1^ix^	112.28 (6)
O1^xvi^—Ba1—O2^xviii^	80.82 (4)	O2—Si1—O1^x^	112.28 (6)
O1^xv^—Ba1—O2^xx^	71.30 (5)	Na1^iv^—O1—Na1^iii^	168.17 (9)
O2^xvii^—Ba1—O2^xix^	119.741 (7)	Na1^iv^—O1—Ba1	89.26 (5)
O2^xvii^—Ba1—O2^xx^	60.259 (7)	Ba1—O1—Na1^iii^	79.67 (4)
O2^xviii^—Ba1—O2^xiii^	60.259 (7)	Zr1—O1—Na1^iv^	100.01 (6)
O2^xvii^—Ba1—O2^xiii^	119.741 (7)	Zr1—O1—Na1^iii^	84.71 (5)
O2—Ba1—O2^xx^	119.740 (7)	Zr1—O1—Ba1	92.27 (5)
O2^xiii^—Ba1—O2^xx^	60.260 (7)	Si1—O1—Na1^iii^	79.82 (5)
O2^xiii^—Ba1—O2^xix^	119.740 (7)	Si1—O1—Na1^iv^	98.38 (6)
O2^xviii^—Ba1—O2^xix^	60.259 (7)	Si1—O1—Ba1	100.86 (7)
O2—Ba1—O2^xiii^	180.0	Si1—O1—Zr1	157.49 (9)
O2^xviii^—Ba1—O2^xx^	119.741 (7)	Na1^xxiii^—O2—Ba1^xxii^	87.07 (4)
O2^xvii^—Ba1—O2	60.259 (7)	Na1^xxiii^—O2—Ba1	87.07 (4)
O2—Ba1—O2^xix^	60.260 (7)	Na1^xxiii^—O2—Ba1^i^	87.07 (4)
O2^xviii^—Ba1—O2	119.741 (7)	Ba1—O2—Ba1^xxii^	119.741 (7)
O2^xvii^—Ba1—O2^xviii^	180.00 (8)	Ba1^i^—O2—Ba1	119.741 (7)
O2^xix^—Ba1—O2^xx^	180.00 (8)	Ba1^i^—O2—Ba1^xxii^	119.741 (7)
Na1^iv^—Zr1—Na1^xx^	77.74 (2)	Si1—O2—Na1^xxiii^	180.0
Na1—Zr1—Na1^xx^	102.26 (2)	Si1—O2—Ba1^i^	92.93 (4)
Na1^xviii^—Zr1—Na1	102.26 (2)	Si1—O2—Ba1^xxii^	92.93 (4)
Na1^iv^—Zr1—Na1^xxi^	102.26 (2)	Si1—O2—Ba1	92.93 (4)
Na1^xxi^—Zr1—Na1^xx^	77.74 (2)		
			
Na1^ii^—Si1—O1—Na1^iv^	80.07 (11)	Ba1^i^—Si1—O1—Zr1	−156.5 (2)
Na1—Si1—O1—Na1^iv^	100.39 (6)	Ba1—Si1—O1—Zr1	124.6 (3)
Na1^iii^—Si1—O1—Na1^iv^	−168.16 (9)	Ba1^xxii^—Si1—O1—Zr1	65.4 (3)
Na1—Si1—O1—Na1^iii^	−91.46 (4)	Ba1—Si1—O2—Ba1^xxii^	−120.0
Na1^iv^—Si1—O1—Na1^iii^	168.16 (9)	Ba1^i^—Si1—O2—Ba1	−120.000 (1)
Na1^ii^—Si1—O1—Na1^iii^	−111.77 (9)	Ba1^xxii^—Si1—O2—Ba1	120.0
Na1—Si1—O1—Ba1	−168.75 (6)	Ba1^i^—Si1—O2—Ba1^xxii^	120.000 (1)
Na1^iv^—Si1—O1—Ba1	90.86 (7)	Ba1^xxii^—Si1—O2—Ba1^i^	−120.0
Na1^iii^—Si1—O1—Ba1	−77.30 (5)	Ba1—Si1—O2—Ba1^i^	120.0
Na1^ii^—Si1—O1—Ba1	170.93 (5)	O1^ix^—Si1—O1—Na1^iii^	−148.17 (6)
Na1^iii^—Si1—O1—Zr1	47.3 (2)	O1^x^—Si1—O1—Na1^iii^	−34.74 (9)
Na1—Si1—O1—Zr1	−44.1 (2)	O1^ix^—Si1—O1—Na1^iv^	43.67 (12)
Na1^ii^—Si1—O1—Zr1	−64.5 (3)	O1^x^—Si1—O1—Na1^iv^	157.10 (5)
Na1^iv^—Si1—O1—Zr1	−144.5 (3)	O1^ix^—Si1—O1—Ba1	134.53 (8)
Na1^iv^—Si1—O2—Ba1	−60.0	O1^x^—Si1—O1—Ba1	−112.04 (9)
Na1^iii^—Si1—O2—Ba1^i^	180.0	O1^ix^—Si1—O1—Zr1	−100.85 (18)
Na1^ii^—Si1—O2—Ba1	180.0	O1^x^—Si1—O1—Zr1	12.6 (3)
Na1^ii^—Si1—O2—Ba1^xxii^	60.0	O1^ix^—Si1—O2—Ba1^xxii^	109.82 (5)
Na1^iv^—Si1—O2—Ba1^xxii^	180.000 (1)	O1^x^—Si1—O2—Ba1	109.81 (5)
Na1^iii^—Si1—O2—Ba1^xxii^	−60.0	O1—Si1—O2—Ba1	−10.18 (5)
Na1^iv^—Si1—O2—Ba1^i^	60.0	O1^ix^—Si1—O2—Ba1^i^	−10.18 (5)
Na1^iii^—Si1—O2—Ba1	60.0	O1^x^—Si1—O2—Ba1^xxii^	−10.19 (5)
Na1^ii^—Si1—O2—Ba1^i^	−60.000 (1)	O1^x^—Si1—O2—Ba1^i^	−130.19 (5)
Ba1—Si1—O1—Na1^iv^	−90.86 (7)	O1—Si1—O2—Ba1^i^	109.82 (5)
Ba1^xxii^—Si1—O1—Na1^iii^	18.11 (8)	O1^ix^—Si1—O2—Ba1	−130.18 (5)
Ba1^i^—Si1—O1—Na1^iv^	−12.01 (8)	O1—Si1—O2—Ba1^xxii^	−130.18 (5)
Ba1—Si1—O1—Na1^iii^	77.30 (5)	O2—Si1—O1—Na1^iv^	−79.61 (6)
Ba1^i^—Si1—O1—Na1^iii^	156.15 (4)	O2—Si1—O1—Na1^iii^	88.54 (4)
Ba1^xxii^—Si1—O1—Na1^iv^	−150.05 (5)	O2—Si1—O1—Ba1	11.25 (6)
Ba1^i^—Si1—O1—Ba1	78.85 (6)	O2—Si1—O1—Zr1	135.9 (2)
Ba1^xxii^—Si1—O1—Ba1	−59.19 (9)		

Symmetry codes: (i) *x*, *y*+1, *z*; (ii) −*x*+1, −*y*+2, −*z*+1; (iii) −*x*+1, −*y*+1, −*z*+1; (iv) −*x*, −*y*+1, −*z*+1; (v) *x*−*y*, *x*, −*z*+1; (vi) *x*−*y*+1, *x*+1, −*z*+1; (vii) *y*, −*x*+*y*, −*z*+1; (viii) *y*, −*x*+*y*+1, −*z*+1; (ix) −*x*+*y*, −*x*+1, *z*; (x) −*y*+1, *x*−*y*+1, *z*; (xi) *x*, *y*, *z*+1; (xii) −*x*+*y*, −*x*, *z*; (xiii) −*x*, −*y*, −*z*; (xiv) −*y*, *x*−*y*, *z*; (xv) *x*−*y*, *x*, −*z*; (xvi) *y*, −*x*+*y*, −*z*; (xvii) −*x*, −*y*+1, −*z*; (xviii) *x*, *y*−1, *z*; (xix) −*x*+1, −*y*+1, −*z*; (xx) *x*−1, *y*−1, *z*; (xxi) −*x*, −*y*, −*z*+1; (xxii) *x*+1, *y*+1, *z*; (xxiii) *x*, *y*, *z*−1.

### Disodium barium hafnium bis(orthosilicate) (II) Crystal data

**Table:**

Na_2_BaHf[SiO_4_]_2_	*D*_x_ = 5.007 Mg m^−^^3^
*M**_r_* = 545.99	Mo *K*α radiation, λ = 0.71073 Å
Trigonal, *P*3	Cell parameters from 7145 reflections
*a* = 5.3889 (2) Å	θ = 2.8–51.0°
*c* = 7.1996 (2) Å	µ = 20.19 mm^−^^1^
*V* = 181.07 (1) Å^3^	*T* = 293 K
*Z* = 1	Plate, colourless
*F*(000) = 242	0.03 × 0.03 × 0.01 mm

### Disodium barium hafnium bis(orthosilicate) (II) Data collection

**Table:**

ROD, Synergy Custom system, HyPix-Arc 150 diffractometer	538 independent reflections
Radiation source: Rotating-anode X-ray tube, Rigaku (Mo) X-ray Source	532 reflections with *I* > 2σ(*I*)
Mirror monochromator	*R*_int_ = 0.045
Detector resolution: 10.0000 pixels mm^-1^	θ_max_ = 35.0°, θ_min_ = 2.8°
ω scans	*h* = −8→7
Absorption correction: gaussian (CrysAlisPro; Rigaku OD, 2023)	*k* = −8→8
*T*_min_ = 0.641, *T*_max_ = 0.883	*l* = −11→11
8609 measured reflections	

### Disodium barium hafnium bis(orthosilicate) (II) Refinement

**Table:**

Refinement on *F*^2^	0 restraints
Least-squares matrix: full	Primary atom site location: dual
*R*[*F*^2^ > 2σ(*F*^2^)] = 0.010	*w* = 1/[σ^2^(*F*_o_^2^) + (0.0035*P*)^2^ + 0.1631*P*] where *P* = (*F*_o_^2^ + 2*F*_c_^2^)/3
*wR*(*F*^2^) = 0.024	(Δ/σ)_max_ < 0.001
*S* = 1.11	Δρ_max_ = 0.61 e Å^−^^3^
538 reflections	Δρ_min_ = −0.60 e Å^−^^3^
24 parameters	

### Disodium barium hafnium bis(orthosilicate) (II) Special details

**Table:**

Geometry. All esds (except the esd in the dihedral angle between two l.s. planes) are estimated using the full covariance matrix. The cell esds are taken into account individually in the estimation of esds in distances, angles and torsion angles; correlations between esds in cell parameters are only used when they are defined by crystal symmetry. An approximate (isotropic) treatment of cell esds is used for estimating esds involving l.s. planes.
Refinement. Refined as a 2-component twin. 1. Twinned data refinement Scales: 0.623 (4) 0.377 (4)

### Disodium barium hafnium bis(orthosilicate) (II) Fractional atomic coordinates and isotropic or equivalent isotropic displacement parameters (Å^2^)

**Table:**

	*x*	*y*	*z*	*U*_iso_*/*U*_eq_	
Na1	0.333333	0.666667	0.7096 (2)	0.0148 (2)	
Ba1	0.000000	0.000000	0.000000	0.01327 (5)	
Hf1	0.000000	0.000000	0.500000	0.00516 (4)	
Si1	0.333333	0.666667	0.24191 (11)	0.00532 (12)	
O1	0.1238 (3)	0.3468 (3)	0.3269 (2)	0.0118 (4)	
O2	0.333333	0.666667	0.0232 (3)	0.0187 (5)	

### Disodium barium hafnium bis(orthosilicate) (II) Atomic displacement parameters (Å^2^)

**Table:**

	*U*^11^	*U*^22^	*U*^33^	*U*^12^	*U*^13^	*U*^23^
Na1	0.0142 (4)	0.0142 (4)	0.0159 (6)	0.00710 (18)	0.000	0.000
Ba1	0.01685 (8)	0.01685 (8)	0.00611 (10)	0.00842 (4)	0.000	0.000
Hf1	0.00461 (5)	0.00461 (5)	0.00626 (7)	0.00231 (2)	0.000	0.000
Si1	0.00477 (18)	0.00477 (18)	0.0064 (3)	0.00239 (9)	0.000	0.000
O1	0.0111 (9)	0.0074 (6)	0.0146 (7)	0.0030 (5)	0.0012 (5)	0.0043 (5)
O2	0.0245 (7)	0.0245 (7)	0.0073 (9)	0.0122 (4)	0.000	0.000

### Disodium barium hafnium bis(orthosilicate) (II) Geometric parameters (Å, º)

**Table:**

Na1—Hf1^i^	3.4579 (6)	Ba1—O1^xv^	2.8687 (16)
Na1—Si1	3.3670 (16)	Ba1—O1^xvi^	2.8687 (16)
Na1—Si1^ii^	3.1308 (2)	Ba1—O2^xvii^	3.1157 (2)
Na1—Si1^iii^	3.1308 (2)	Ba1—O2^xviii^	3.1158 (2)
Na1—Si1^iv^	3.1308 (2)	Ba1—O2^xiv^	3.1158 (2)
Na1—O1^v^	2.4423 (15)	Ba1—O2^xix^	3.1157 (2)
Na1—O1^iv^	2.4423 (15)	Ba1—O2	3.1158 (2)
Na1—O1^vi^	2.4423 (15)	Ba1—O2^xx^	3.1158 (2)
Na1—O1^vii^	2.9738 (15)	Hf1—O1^v^	2.0600 (15)
Na1—O1^viii^	2.9738 (15)	Hf1—O1^xxi^	2.0600 (15)
Na1—O1^iii^	2.9738 (15)	Hf1—O1^xiii^	2.0600 (15)
Na1—O1	3.145 (2)	Hf1—O1	2.0600 (15)
Na1—O1^ix^	3.145 (2)	Hf1—O1^vii^	2.0600 (15)
Na1—O1^x^	3.145 (2)	Hf1—O1^xvi^	2.0600 (15)
Na1—O2^xi^	2.258 (3)	Si1—O1^x^	1.6354 (16)
Ba1—O1^xii^	2.8687 (16)	Si1—O1	1.6354 (16)
Ba1—O1^xiii^	2.8687 (16)	Si1—O1^ix^	1.6354 (16)
Ba1—O1^xiv^	2.8687 (16)	Si1—O2	1.575 (2)
Ba1—O1	2.8687 (16)		
			
Si1^iii^—Na1—Hf1^i^	160.53 (5)	Na1^xix^—Hf1—Na1^iv^	180.0
Si1^ii^—Na1—Hf1^i^	66.522 (10)	Na1^xix^—Hf1—Na1^xxi^	77.62 (3)
Si1^iv^—Na1—Hf1^i^	66.522 (10)	Na1^xix^—Hf1—Na1^iii^	77.62 (3)
Si1—Na1—Hf1^i^	64.13 (2)	Na1—Hf1—Na1^xxi^	180.0
Si1^ii^—Na1—Si1^iii^	118.773 (12)	Na1—Hf1—Na1^iii^	77.62 (3)
Si1^ii^—Na1—Si1^iv^	118.773 (12)	Na1^iv^—Hf1—Na1^xx^	77.62 (3)
Si1^iv^—Na1—Si1^iii^	118.773 (12)	Na1^xxi^—Hf1—Na1^iii^	102.38 (3)
Si1^ii^—Na1—Si1	96.40 (3)	Na1^xxi^—Hf1—Na1^xx^	77.62 (3)
Si1^iv^—Na1—Si1	96.40 (3)	Na1^xix^—Hf1—Na1	102.38 (3)
Si1^iii^—Na1—Si1	96.40 (3)	Na1^iv^—Hf1—Na1	77.62 (3)
O1^iii^—Na1—Hf1^i^	138.33 (5)	O1^vii^—Hf1—Na1^xix^	116.29 (5)
O1^viii^—Na1—Hf1^i^	36.38 (3)	O1^xvi^—Hf1—Na1^xx^	63.71 (5)
O1^vii^—Na1—Hf1^i^	86.70 (3)	O1^v^—Hf1—Na1	44.07 (4)
O1^iv^—Na1—Hf1^i^	35.92 (4)	O1^xiii^—Hf1—Na1^xix^	63.71 (5)
O1^v^—Na1—Hf1^i^	137.65 (5)	O1^xxi^—Hf1—Na1^xx^	58.91 (4)
O1^vi^—Na1—Hf1^i^	85.99 (4)	O1^xxi^—Hf1—Na1^xix^	44.07 (4)
O1^iv^—Na1—Si1^ii^	92.15 (4)	O1^vii^—Hf1—Na1^iv^	63.71 (5)
O1^iii^—Na1—Si1^iii^	30.94 (3)	O1^xxi^—Hf1—Na1	116.29 (5)
O1^iv^—Na1—Si1^iii^	148.71 (4)	O1^vii^—Hf1—Na1^xx^	44.07 (4)
O1^vi^—Na1—Si1^ii^	31.12 (4)	O1^v^—Hf1—Na1^iv^	121.09 (4)
O1^viii^—Na1—Si1	84.93 (4)	O1^xxi^—Hf1—Na1^iv^	135.93 (4)
O1^v^—Na1—Si1^ii^	148.71 (4)	O1^xvi^—Hf1—Na1^iii^	116.29 (5)
O1^viii^—Na1—Si1^iii^	148.92 (3)	O1^vii^—Hf1—Na1^iii^	135.93 (4)
O1^viii^—Na1—Si1^ii^	30.94 (3)	O1—Hf1—Na1^iii^	58.91 (4)
O1^vi^—Na1—Si1	83.83 (5)	O1^v^—Hf1—Na1^xx^	116.29 (5)
O1^vii^—Na1—Si1^ii^	148.92 (3)	O1^xvi^—Hf1—Na1^xxi^	44.07 (4)
O1^iii^—Na1—Si1	84.93 (4)	O1—Hf1—Na1^xx^	121.09 (4)
O1^iii^—Na1—Si1^ii^	91.76 (3)	O1—Hf1—Na1^xxi^	116.29 (5)
O1^v^—Na1—Si1^iii^	31.12 (4)	O1^xiii^—Hf1—Na1^xx^	135.93 (4)
O1^iv^—Na1—Si1^iv^	31.12 (4)	O1^vii^—Hf1—Na1^xxi^	121.09 (4)
O1^vii^—Na1—Si1^iii^	91.76 (3)	O1^xxi^—Hf1—Na1^iii^	121.09 (4)
O1^vi^—Na1—Si1^iv^	148.71 (4)	O1^xiii^—Hf1—Na1^xxi^	58.91 (4)
O1^iv^—Na1—Si1	83.83 (5)	O1—Hf1—Na1^xix^	135.93 (4)
O1^v^—Na1—Si1^iv^	92.15 (4)	O1^xxi^—Hf1—Na1^xxi^	63.71 (5)
O1^v^—Na1—Si1	83.83 (5)	O1^v^—Hf1—Na1^iii^	63.71 (5)
O1^viii^—Na1—Si1^iv^	91.76 (3)	O1^xvi^—Hf1—Na1^iv^	58.91 (4)
O1^vii^—Na1—Si1	84.93 (4)	O1—Hf1—Na1^iv^	44.07 (4)
O1^vii^—Na1—Si1^iv^	30.94 (3)	O1^v^—Hf1—Na1^xxi^	135.93 (4)
O1^iii^—Na1—Si1^iv^	148.92 (3)	O1^xiii^—Hf1—Na1^iii^	44.07 (4)
O1^vi^—Na1—Si1^iii^	92.15 (4)	O1^xvi^—Hf1—Na1	135.93 (4)
O1^v^—Na1—O1^iii^	57.02 (7)	O1^v^—Hf1—Na1^xix^	58.91 (4)
O1^iv^—Na1—O1^viii^	62.36 (7)	O1—Hf1—Na1	63.71 (5)
O1^vi^—Na1—O1^vii^	168.44 (9)	O1^xvi^—Hf1—Na1^xix^	121.09 (4)
O1^v^—Na1—O1^vii^	62.36 (7)	O1^vii^—Hf1—Na1	58.91 (4)
O1^v^—Na1—O1^viii^	168.44 (9)	O1^xiii^—Hf1—Na1	121.09 (4)
O1^viii^—Na1—O1^iii^	119.229 (13)	O1^xiii^—Hf1—Na1^iv^	116.29 (5)
O1^vi^—Na1—O1^v^	118.859 (19)	O1^v^—Hf1—O1^xxi^	87.19 (7)
O1^vii^—Na1—O1^iii^	119.229 (13)	O1^v^—Hf1—O1^vii^	87.19 (7)
O1^vi^—Na1—O1^viii^	57.02 (7)	O1—Hf1—O1^xiii^	87.19 (7)
O1^iv^—Na1—O1^iii^	168.44 (9)	O1^xvi^—Hf1—O1^xxi^	92.81 (7)
O1^iv^—Na1—O1^vii^	57.02 (7)	O1^v^—Hf1—O1^xiii^	92.81 (7)
O1^vi^—Na1—O1^iii^	62.36 (7)	O1—Hf1—O1^xxi^	180.0
O1^iv^—Na1—O1^vi^	118.859 (19)	O1^xvi^—Hf1—O1	87.19 (7)
O1^iv^—Na1—O1^v^	118.859 (19)	O1^vii^—Hf1—O1^xxi^	87.19 (7)
O1^viii^—Na1—O1^vii^	119.229 (13)	O1—Hf1—O1^vii^	92.81 (7)
O2^xi^—Na1—Hf1^i^	115.87 (2)	O1^xiii^—Hf1—O1^xxi^	92.81 (7)
O2^xi^—Na1—Si1^iii^	83.60 (3)	O1^xvi^—Hf1—O1^xiii^	87.19 (7)
O2^xi^—Na1—Si1	180.0	O1^v^—Hf1—O1^xvi^	180.0
O2^xi^—Na1—Si1^iv^	83.60 (3)	O1^vii^—Hf1—O1^xiii^	180.0
O2^xi^—Na1—Si1^ii^	83.60 (3)	O1^v^—Hf1—O1	92.81 (7)
O2^xi^—Na1—O1^vii^	95.07 (4)	O1^xvi^—Hf1—O1^vii^	92.81 (7)
O2^xi^—Na1—O1^v^	96.17 (5)	Na1^ii^—Si1—Na1^iv^	118.773 (12)
O2^xi^—Na1—O1^viii^	95.07 (4)	Na1^iii^—Si1—Na1	83.60 (3)
O2^xi^—Na1—O1^vi^	96.17 (5)	Na1^iv^—Si1—Na1	83.60 (3)
O2^xi^—Na1—O1^iv^	96.17 (5)	Na1^iv^—Si1—Na1^iii^	118.772 (12)
O2^xi^—Na1—O1^iii^	95.07 (4)	Na1^ii^—Si1—Na1	83.60 (3)
O1^xiii^—Ba1—O1	59.36 (5)	Na1^ii^—Si1—Na1^iii^	118.772 (12)
O1^xiv^—Ba1—O1	180.0	Na1^ii^—Si1—Ba1^i^	67.724 (16)
O1^xv^—Ba1—O1^xvi^	120.64 (5)	Na1^iv^—Si1—Ba1^xxii^	157.16 (4)
O1^xii^—Ba1—O1^xvi^	180.00 (8)	Na1—Si1—Ba1	119.240 (11)
O1^xiv^—Ba1—O1^xvi^	120.64 (5)	Na1^iii^—Si1—Ba1^i^	157.16 (4)
O1^xiv^—Ba1—O1^xii^	59.36 (5)	Na1—Si1—Ba1^xxii^	119.240 (11)
O1^xiii^—Ba1—O1^xvi^	59.36 (5)	Na1—Si1—Ba1^i^	119.240 (11)
O1^xv^—Ba1—O1^xii^	59.36 (5)	Na1^ii^—Si1—Ba1^xxii^	67.723 (16)
O1^xiii^—Ba1—O1^xii^	120.64 (5)	Na1^ii^—Si1—Ba1	157.16 (4)
O1^xv^—Ba1—O1	120.64 (5)	Na1^iii^—Si1—Ba1^xxii^	67.723 (16)
O1^xiv^—Ba1—O1^xv^	59.36 (5)	Na1^iv^—Si1—Ba1	67.723 (16)
O1^xii^—Ba1—O1	120.64 (5)	Na1^iii^—Si1—Ba1	67.723 (16)
O1^xiv^—Ba1—O1^xiii^	120.64 (5)	Na1^iv^—Si1—Ba1^i^	67.724 (16)
O1^xv^—Ba1—O1^xiii^	180.00 (10)	Ba1—Si1—Ba1^xxii^	98.169 (14)
O1^xvi^—Ba1—O1	59.36 (5)	Ba1^i^—Si1—Ba1	98.169 (14)
O1^xii^—Ba1—O2^xviii^	52.61 (5)	Ba1^i^—Si1—Ba1^xxii^	98.169 (14)
O1—Ba1—O2	52.61 (5)	O1^ix^—Si1—Na1^ii^	50.52 (5)
O1^xv^—Ba1—O2^xviii^	108.55 (5)	O1^x^—Si1—Na1	68.03 (6)
O1—Ba1—O2^xviii^	80.96 (5)	O1—Si1—Na1^ii^	149.93 (7)
O1^xii^—Ba1—O2^xiv^	108.55 (5)	O1^x^—Si1—Na1^iv^	149.94 (7)
O1^xv^—Ba1—O2^xx^	71.45 (5)	O1^ix^—Si1—Na1^iv^	69.22 (5)
O1^xiii^—Ba1—O2	99.04 (5)	O1^ix^—Si1—Na1^iii^	149.94 (7)
O1^xii^—Ba1—O2^xx^	127.39 (5)	O1—Si1—Na1	68.03 (6)
O1^xv^—Ba1—O2^xiv^	99.04 (5)	O1—Si1—Na1^iii^	69.22 (5)
O1—Ba1—O2^xx^	99.04 (5)	O1^x^—Si1—Na1^ii^	69.22 (5)
O1—Ba1—O2^xiv^	127.39 (5)	O1^x^—Si1—Na1^iii^	50.51 (5)
O1—Ba1—O2^xvii^	71.45 (5)	O1—Si1—Na1^iv^	50.51 (5)
O1^xv^—Ba1—O2	80.96 (5)	O1^ix^—Si1—Na1	68.03 (6)
O1^xiv^—Ba1—O2^xix^	71.45 (5)	O1—Si1—Ba1^xxii^	134.78 (5)
O1^xii^—Ba1—O2	71.45 (5)	O1^ix^—Si1—Ba1^i^	52.13 (6)
O1^xv^—Ba1—O2^xix^	127.39 (5)	O1^ix^—Si1—Ba1^xxii^	117.23 (5)
O1^xiv^—Ba1—O2^xiv^	52.61 (5)	O1^x^—Si1—Ba1^xxii^	52.13 (6)
O1^xiii^—Ba1—O2^xix^	52.61 (5)	O1—Si1—Ba1^i^	117.23 (5)
O1^xiii^—Ba1—O2^xiv^	80.96 (5)	O1^x^—Si1—Ba1	117.23 (5)
O1^xii^—Ba1—O2^xix^	80.96 (5)	O1—Si1—Ba1	52.13 (6)
O1^xvi^—Ba1—O2^xiv^	71.45 (5)	O1^x^—Si1—Ba1^i^	134.78 (5)
O1^xvi^—Ba1—O2^xix^	99.04 (5)	O1^ix^—Si1—Ba1	134.78 (6)
O1^xiv^—Ba1—O2^xviii^	99.04 (5)	O1^ix^—Si1—O1	106.86 (7)
O1—Ba1—O2^xix^	108.55 (5)	O1—Si1—O1^x^	106.86 (7)
O1^xiii^—Ba1—O2^xviii^	71.45 (5)	O1^ix^—Si1—O1^x^	106.86 (7)
O1^xiv^—Ba1—O2	127.39 (5)	O2—Si1—Na1	180.0
O1^xvi^—Ba1—O2^xviii^	127.39 (5)	O2—Si1—Na1^iii^	96.40 (3)
O1^xiv^—Ba1—O2^xvii^	108.55 (5)	O2—Si1—Na1^iv^	96.40 (3)
O1^xiv^—Ba1—O2^xx^	80.96 (5)	O2—Si1—Na1^ii^	96.40 (3)
O1^xv^—Ba1—O2^xvii^	52.61 (5)	O2—Si1—Ba1	60.760 (11)
O1^xiii^—Ba1—O2^xx^	108.55 (5)	O2—Si1—Ba1^xxii^	60.760 (11)
O1^xiii^—Ba1—O2^xvii^	127.39 (5)	O2—Si1—Ba1^i^	60.760 (11)
O1^xvi^—Ba1—O2^xx^	52.61 (5)	O2—Si1—O1	111.97 (6)
O1^xii^—Ba1—O2^xvii^	99.04 (5)	O2—Si1—O1^x^	111.97 (6)
O1^xvi^—Ba1—O2^xvii^	80.96 (5)	O2—Si1—O1^ix^	111.97 (6)
O1^xvi^—Ba1—O2	108.55 (5)	Na1^iv^—O1—Na1^iii^	168.44 (9)
O2^xvii^—Ba1—O2^xviii^	119.716 (8)	Na1^iv^—O1—Ba1	89.42 (6)
O2^xvii^—Ba1—O2^xiv^	119.716 (8)	Ba1—O1—Na1^iii^	79.80 (5)
O2^xiv^—Ba1—O2^xx^	60.285 (8)	Hf1—O1—Na1^iii^	84.71 (5)
O2^xix^—Ba1—O2^xviii^	60.284 (8)	Hf1—O1—Na1^iv^	100.01 (6)
O2^xix^—Ba1—O2^xx^	119.716 (8)	Hf1—O1—Ba1	92.35 (6)
O2—Ba1—O2^xviii^	60.285 (8)	Si1—O1—Na1^iv^	98.37 (7)
O2^xvii^—Ba1—O2^xix^	180.0	Si1—O1—Na1^iii^	79.84 (6)
O2^xiv^—Ba1—O2^xviii^	119.715 (8)	Si1—O1—Ba1	101.12 (7)
O2^xvii^—Ba1—O2^xx^	60.284 (8)	Si1—O1—Hf1	157.27 (10)
O2—Ba1—O2^xiv^	180.0	Na1^xxiii^—O2—Ba1^xxii^	86.93 (4)
O2—Ba1—O2^xx^	119.715 (8)	Na1^xxiii^—O2—Ba1^i^	86.93 (4)
O2^xvii^—Ba1—O2	60.285 (8)	Na1^xxiii^—O2—Ba1	86.93 (4)
O2^xix^—Ba1—O2^xiv^	60.284 (8)	Ba1^i^—O2—Ba1^xxii^	119.715 (8)
O2^xix^—Ba1—O2	119.715 (8)	Ba1—O2—Ba1^xxii^	119.715 (8)
O2^xviii^—Ba1—O2^xx^	180.0	Ba1^i^—O2—Ba1	119.715 (8)
Na1^iv^—Hf1—Na1^iii^	102.38 (3)	Si1—O2—Na1^xxiii^	180.0
Na1^iv^—Hf1—Na1^xxi^	102.38 (3)	Si1—O2—Ba1	93.07 (4)
Na1—Hf1—Na1^xx^	102.38 (3)	Si1—O2—Ba1^i^	93.07 (4)
Na1^xx^—Hf1—Na1^iii^	180.0	Si1—O2—Ba1^xxii^	93.07 (4)
Na1^xix^—Hf1—Na1^xx^	102.38 (3)		
			
Na1^ii^—Si1—O1—Na1^iv^	79.72 (11)	Ba1^i^—Si1—O1—Hf1	−156.1 (2)
Na1—Si1—O1—Na1^iii^	−91.40 (4)	Ba1—Si1—O1—Hf1	125.3 (3)
Na1^iv^—Si1—O1—Na1^iii^	168.43 (9)	Ba1^xxii^—Si1—O1—Hf1	66.5 (3)
Na1^iii^—Si1—O1—Na1^iv^	−168.43 (9)	Ba1^xxii^—Si1—O2—Ba1^i^	−120.0
Na1^ii^—Si1—O1—Na1^iii^	−111.84 (10)	Ba1^xxii^—Si1—O2—Ba1	120.0
Na1—Si1—O1—Na1^iv^	100.17 (6)	Ba1^i^—Si1—O2—Ba1	−120.000 (1)
Na1^ii^—Si1—O1—Ba1	170.78 (6)	Ba1—Si1—O2—Ba1^i^	120.0
Na1—Si1—O1—Ba1	−168.78 (6)	Ba1—Si1—O2—Ba1^xxii^	−120.0
Na1^iii^—Si1—O1—Ba1	−77.38 (5)	Ba1^i^—Si1—O2—Ba1^xxii^	120.0
Na1^iv^—Si1—O1—Ba1	91.06 (7)	O1^ix^—Si1—O1—Na1^iii^	−148.46 (7)
Na1^iv^—Si1—O1—Hf1	−143.7 (3)	O1^ix^—Si1—O1—Na1^iv^	43.11 (12)
Na1^iii^—Si1—O1—Hf1	47.9 (2)	O1^x^—Si1—O1—Na1^iv^	157.23 (5)
Na1^ii^—Si1—O1—Hf1	−63.9 (3)	O1^x^—Si1—O1—Na1^iii^	−34.34 (9)
Na1—Si1—O1—Hf1	−43.5 (2)	O1^ix^—Si1—O1—Ba1	134.17 (9)
Na1^ii^—Si1—O2—Ba1^i^	−60.000 (1)	O1^x^—Si1—O1—Ba1	−111.72 (10)
Na1^iv^—Si1—O2—Ba1	−60.0	O1^ix^—Si1—O1—Hf1	−100.56 (19)
Na1^ii^—Si1—O2—Ba1^xxii^	60.0	O1^x^—Si1—O1—Hf1	13.6 (3)
Na1^iii^—Si1—O2—Ba1^xxii^	−60.0	O1^ix^—Si1—O2—Ba1^i^	−10.14 (6)
Na1^iii^—Si1—O2—Ba1	60.0	O1^x^—Si1—O2—Ba1^i^	−130.14 (6)
Na1^iii^—Si1—O2—Ba1^i^	180.0	O1^ix^—Si1—O2—Ba1	−130.14 (6)
Na1^iv^—Si1—O2—Ba1^xxii^	180.000 (1)	O1—Si1—O2—Ba1	−10.14 (6)
Na1^ii^—Si1—O2—Ba1	180.0	O1^x^—Si1—O2—Ba1	109.86 (6)
Na1^iv^—Si1—O2—Ba1^i^	60.0	O1—Si1—O2—Ba1^xxii^	−130.14 (6)
Ba1—Si1—O1—Na1^iv^	−91.06 (7)	O1^x^—Si1—O2—Ba1^xxii^	−10.14 (6)
Ba1—Si1—O1—Na1^iii^	77.38 (5)	O1^ix^—Si1—O2—Ba1^xxii^	109.86 (6)
Ba1^i^—Si1—O1—Na1^iii^	155.97 (4)	O1—Si1—O2—Ba1^i^	109.86 (6)
Ba1^xxii^—Si1—O1—Na1^iv^	−149.85 (6)	O2—Si1—O1—Na1^iv^	−79.83 (6)
Ba1^i^—Si1—O1—Na1^iv^	−12.46 (9)	O2—Si1—O1—Na1^iii^	88.60 (4)
Ba1^xxii^—Si1—O1—Na1^iii^	18.58 (9)	O2—Si1—O1—Ba1	11.22 (6)
Ba1^i^—Si1—O1—Ba1	78.60 (6)	O2—Si1—O1—Hf1	136.5 (2)
Ba1^xxii^—Si1—O1—Ba1	−58.80 (9)		

Symmetry codes: (i) *x*, *y*+1, *z*; (ii) −*x*+1, −*y*+2, −*z*+1; (iii) −*x*+1, −*y*+1, −*z*+1; (iv) −*x*, −*y*+1, −*z*+1; (v) *y*, −*x*+*y*, −*z*+1; (vi) *x*−*y*+1, *x*+1, −*z*+1; (vii) *x*−*y*, *x*, −*z*+1; (viii) *y*, −*x*+*y*+1, −*z*+1; (ix) −*x*+*y*, −*x*+1, *z*; (x) −*y*+1, *x*−*y*+1, *z*; (xi) *x*, *y*, *z*+1; (xii) *y*, −*x*+*y*, −*z*; (xiii) −*x*+*y*, −*x*, *z*; (xiv) −*x*, −*y*, −*z*; (xv) *x*−*y*, *x*, −*z*; (xvi) −*y*, *x*−*y*, *z*; (xvii) −*x*, −*y*+1, −*z*; (xviii) −*x*+1, −*y*+1, −*z*; (xix) *x*, *y*−1, *z*; (xx) *x*−1, *y*−1, *z*; (xxi) −*x*, −*y*, −*z*+1; (xxii) *x*+1, *y*+1, *z*; (xxiii) *x*, *y*, *z*−1.
